# Diagnostic accuracy of loop-mediated isothermal amplification (LAMP) for screening malaria in peripheral and placental blood samples from pregnant women in Colombia

**DOI:** 10.1186/s12936-018-2403-5

**Published:** 2018-07-13

**Authors:** Ana María Vásquez, Lina Zuluaga, Alberto Tobón, Maritza Posada, Gabriel Vélez, Iveth J. González, Ana Campillo, Xavier Ding

**Affiliations:** 1 Grupo Malaria, Facultad de Medicina, Universidad de Antioquia, Carrera 53 No. 61–30, Lab 610, Medellín, Colombia; 2 FIND, Geneva, Switzerland

**Keywords:** Malaria in pregnancy, Diagnostics, Rapid diagnostic test, Light microscopy, Loop mediated isothermal amplification, Polymerase chain reaction

## Abstract

**Background:**

Pregnant women frequently show low-density *Plasmodium* infections that require more sensitive methods for accurate diagnosis and early treatment of malaria. This is particularly relevant in low-malaria transmission areas, where intermittent preventive treatment is not recommended. Molecular methods, such as polymerase chain reaction (PCR) are highly sensitive, but require sophisticated equipment and advanced training. Instead, loop mediated isothermal amplification (LAMP) provides an opportunity for molecular detection of malaria infections in remote endemic areas, outside a reference laboratory. The aim of the study is to evaluate the performance of LAMP for the screening of malaria in pregnant women in Colombia.

**Methods:**

This is a nested prospective study that uses data and samples from a larger cross-sectional project conducted from May 2016 to January 2017 in three Colombian endemic areas (El Bagre, Quibdó, and Tumaco). A total of 531 peripheral and placental samples from pregnant women self-presenting at local hospitals for antenatal care visits, at delivery or seeking medical care for suspected malaria were collected. Samples were analysed for *Plasmodium* parasites by light microscopy (LM), rapid diagnostic test (RDT) and LAMP. Diagnostic accuracy endpoints (sensitivity, specificity, predictive values, and kappa scores) of LM, RDT and LAMP were compared with nested PCR (nPCR) as the reference standard.

**Results:**

In peripheral samples, LAMP showed an improved sensitivity (100.0%) when compared with LM 79.5% and RDT 76.9% (*p *< 0.01), particularly in afebrile women, for which LAMP sensitivity was two-times higher than LM and RDT. Overall agreement among LAMP and nPCR was high (kappa value = 1.0). Specificity was similar in all tests (100%). In placental blood, LAMP evidenced a four-fold improvement in sensitivity (88.9%) when compared with LM and RDT (22.2%), being the only method, together with nPCR, able to detect placental infections in peripheral blood.

**Conclusions:**

LAMP is a simple, rapid and accurate molecular tool for detecting gestational and placental malaria, being able to overcome the limited sensitivity of LM and RDT. These findings could guide maternal health programs in low-transmission settings to integrate LAMP in their surveillance systems for the active detection of low-density infections and asymptomatic malaria cases.

## Background

Pregnant women are especially susceptible to *Plasmodium* infections and have the risk of developing severe disease and birth complications. These might include maternal anaemia, intra-uterine growth retardation, infant low birth weight, prematurity, miscarriage and stillbirth [1–4]. In the American continent, about three million of women are exposed to the risk of infection [5]. Colombia is the third contributor to overall malaria cases in Latin America, the majority of them being caused by *Plasmodium falciparum* (60%) followed by *Plasmodium vivax* (40%) [6]. Besides the increasing recognition of *P. vivax* deleterious impact on pregnant women’s health [2], *P. falciparum* causes the most severe consequences [2, 7, 8].

Diagnosis of *P. falciparum* during pregnancy remains challenging due to sequestration of parasites in the placenta and their subsequent circulation at low-density levels in peripheral blood [9–11]. In addition, low-density *P. vivax* infections are common in low-malaria transmission regions such as Colombia [2, 12–14], hampering the detection of these parasites by conventional diagnostic tools. Accurate diagnosis and early treatment of malaria in pregnancy (MiP) is therefore crucial for preventing malaria-related pregnancy complications, particularly in low-malaria transmission areas, where intermittent preventive treatment is not recommended [15].

Although light microscopy (LM) remains the standard of practice for malaria diagnosis in clinical settings [6], this method is time-consuming, requires well-trained personnel and does not adequately detect low parasitaemia [15, 16]. Alternatives to LM include rapid diagnostic tests (RDTs) [6], which are easy to use and have facilitated access to malaria diagnosis outside health facilities in peripheral communities. Nonetheless, conventional RDTs are not significantly more sensitive than LM and, similarly, cannot detect the low-level blood-stage malaria infections that can otherwise be identified by molecular methods such as nucleic acid amplification techniques (NAATs) [17].

Molecular techniques, such as polymerase chain reaction (PCR), have the potential to provide more sensitive estimates of maternal infection (as low as 0.1 parasite/μL of whole blood) [18]. Nevertheless, PCR requires sophisticated laboratory conditions, specialized equipment, advanced staff training, relatively long time-to-results and high costs, which are not always feasible in resource-limited settings [18, 19]. Loop mediated isothermal amplification (LAMP) is a NAAT that displays similar sensitivity to PCR (down to 1 parasite/μL of blood) [20] and is an optimal alternative to PCR-based tests as it can be relatively easily deployed outside reference laboratories. Moreover, LAMP presents many operational advantages over PCR, including minimal equipment, shorter time-to-result (30–60 min), lower cost and, from a technical point of view, less complexity [21–23].

The commercially available Loopamp MALARIA Pan/Pf detection kit (Eiken Chemical Co., Tokyo, Japan) consists of reaction tubes with ready-made vacuum-dried reagents for the detection of *P. falciparum* (Pf-LAMP) or *Plasmodium* spp. (Pan-LAMP) [21]. This diagnostic kit has been evaluated in several studies, showing accurate detection of *Plasmodium* infection in both, reference laboratories and field settings [22, 24–27], as well as in low-density infections in asymptomatic and symptomatic patients [12, 24, 26, 27]. Recently, the performance of the LAMP kit for the screening of MiP has been evaluated in high-transmission areas in Africa, showing the usefulness of this method for the diagnosis of malaria in venous [28, 29] and placental blood samples [29, 30]. However, the accuracy of LAMP for the diagnosis of MiP in malaria low-endemic settings remains unknown.

Altogether, sensitive methods such as LAMP could be useful for screening malaria in pregnant women attending antenatal care (ANC) visits and at delivery in local hospitals located in remote endemic areas. This is particularly true in low-malaria transmission settings where it is increasingly recognized that a great proportion of asymptomatic cases are caused by low-density infections [31], which potentially remain as reservoirs for malaria transmission [32–37]. The overall purpose of this study was to evaluate the performance of LAMP for screening malaria in peripheral and placental blood samples from pregnant women living in three Colombian malaria endemic municipalities. The accuracy of LAMP for detecting febrile and afebrile infections by *Plasmodium* spp. in pregnant women, as well as the feasibility of performing this test outside a reference laboratory (local hospital) under minimum infrastructure conditions were also evaluated.

## Methods

### Study design and participants

This is a prospective descriptive study that was part of a larger cross-sectional project aiming to characterize asymptomatic infections in Colombian pregnant women and to assess the impact of these infections in malaria-related adverse pregnancy outcomes. From May 2016 to January 2017, 656 pregnant women self-presenting at local hospitals for ANC visit, at delivery or seeking medical care for suspected malaria were recruited consecutively. Each participant was enrolled only once in one of the two study groups (ANC or delivery). Women, aged ≥ 15 years old, at any gestational age, and living in peri-urban municipality areas or rural areas with malaria transmission were considered eligible. Only 531 women, who provided the required laboratory sample for RDT, LM, LAMP and nPCR testing, were included in the current study.

### Study area

**Fig. 1:**
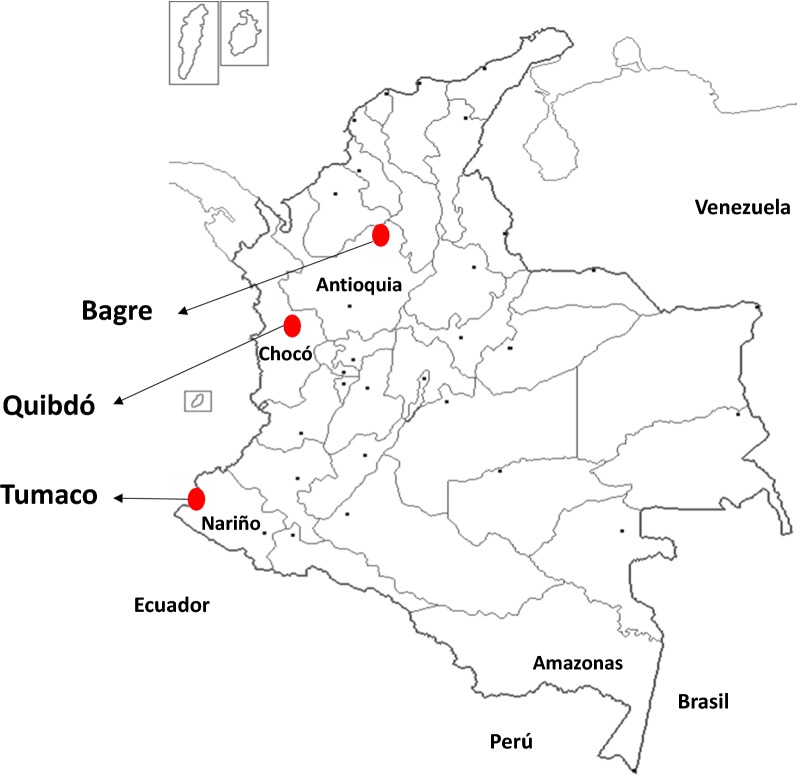
Study area. Map of Colombia showing the three study sites in red dots: Bagre, Quibdó and Tumaco

The study was conducted in three Colombian malaria-endemic municipalities with high transmission rates according to the annual parasite index (API) older than 10 (number of cases per 1000 inhabitants) and different proportion of *P. falciparum* and *P. vivax* (Fig. 1). El Bagre is a municipality located in Antioquia´s Department, in the northwest of Colombia, with *P. vivax* as the predominant *Plasmodium* spp. (70%). El Bagre is located in Antioquia’s Department, in the northwest of Colombia, with *P. vivax* as the predominant *Plasmodium* specie (spp.) (70%) and API of 25.2 in 2016. The municipality of Quibdó is located in the pacific region, in the Chocó’s Department, in the west of Colombia, with *P. falciparum* as the predominant parasite (80%) and API of 100.8 in 2016. Tumaco is located in the pacific coast in Nariño´s Department, in the southwest of the country near the border with Ecuador, where the predominant specie is *P. falciparum* (90%) and API of 16 in 2016.

The study locations were selected based on the incidence and number of symptomatic cases in general population reported during previous years in Colombia [38]. In Colombia, Intermittent preventive treatment (IPTp) is not recommended and national guidelines for diagnosis and treatment of malaria recommend screening for malaria by microscopy during ANC visits to pregnant women living in endemic municipalities [39].

### Data and sample collection

Socio-demographic profiles, obstetric history and other clinical data (history of fever and drug use) were gathered at local hospitals using an interview-based questionnaire. Axillary temperature was also recorded using a digital thermometer. A total of 8 mL of peripheral blood was collected from each participant by venepuncture, using heparin tubes. When pregnant women were enrolled at delivery, 8 mL of placental blood was also collected.

### Sample processing

Blood samples were transferred to the local hospital’s laboratory. Haemoglobin levels were assessed using a HemoCue (Reference 201+, Hemocue AB, Sweden). Thick and thin blood smears (80 µL of blood) were prepared. LM readings, RDT testing (5 µL), DNA extraction (60 µL), as well as LAMP assay were also carried out on-site. Women with positive LM or RDT results received free anti-malarial treatment according to national treatment guidelines. In addition, 200 µL of blood were spotted on Whatman filter paper #3 (Fisher, Ref 1003-917), air-dried, and stored at room temperature in sealed bags with desiccant until transported to the reference laboratory at the Universidad de Antioquia in Medellín for nPCR testing.

### Diagnostic test procedures

The SD Bioline Malaria antigen Pf/Pv (Standard Diagnositcs, Korea, 05FK80) was used by trained staff according to manufacturer’s instructions. This test was selected based on its ability to distinguish between *P. falciparum*, *P. vivax* and mixed infections, and because it is one of the most commonly used RDTs in Colombia. Moreover, this tool is one of the best performing RDTs as reported in the World Health Organization-FIND RDT evaluation programme [40].

Field-stained thick and thin blood slides were read by an expert malaria microscopist according to national guidelines [41]. Parasitaemia was estimated against 200 leukocytes (8000 leukocytes/μL, standard value) and was expressed as parasites/μL (p/μL). *Plasmodium falciparum* parasitaemia was calculated counting ring forms, while *P. vivax* parasitaemia was calculated counting all asexual forms. A sample was considered negative if after the examination of 200 microscopic fields at 100 × magnification, no parasites were observed. As a quality control, a second reading was performed in all PCR positive samples and 10% of PCR negative samples. Discrepant results (positive vs. negative, parasitaemia difference > 50% or different species) were resolved by a third reading. The final parasitaemia was the average of all readings, calculated as previously described.

LAMP kits were used according to FIND’s standard operating procedures [20] and manufacturer’s instructions. All samples were processed to obtain DNA within same day of sampling, briefly, parasite DNA was extracted using the “boil and spin” method [20], where 60 μL of heparin-blood was dispensed into a 1.5 mL tube containing 60 μL of DNA extraction buffer (400 mM NaCl, 40 mM Tris pH 6.5, and 0.4% sodium dodecyl sulfate), mixed by flicking, heated for 5 min at 95 °C in a heat-block and then centrifuged at 10,000*g* for 3 min. The supernatant (30 μL) was transferred into a tube containing 345 μL of sterile water. After blood sample processing, 30 μL of diluted DNA elution were added to the Pan-LAMP reaction tubes and the reagents resuspended according to the manufacturer’s instructions. Samples were incubated for 40 min at 65 °C in a heat-block, followed by 5 min at 80 °C to stop the reaction. DNA amplification was detected by naked eye based on the fluorescence observed within the reaction mix when using an ultraviolet (UV) lamp. The equivalent blood sample input for LAMP was approximately 1.2 μL of whole blood. All samples positive for Pan-LAMP were then retested using Pf-LAMP specific kits. Positive and negative controls were included in each LAMP assay run. Three research assistants, one in each site, were trained over 2 days on LAMP procedures for sample processing, amplification and detection.

DNA was extracted from half blood-spot filter (approximately 30 µL of blood) using QIAamp DNA Mini Kit (Qiagen, Germany, Ref 51306), according to manufacturer’s instructions. Nested PCR was performed as a two-step procedure, using 2 μl of DNA template and following the protocol described by Singh et al. with minor modifications [42]. This protocol consists of a universal PCR followed by nested species-specific PCR to detect the 18S ribosomal gene of *P. falciparum*, *P. vivax* and *Plasmodium malariae*. The equivalent blood sample input for PCR was approximately 0.6 μL of whole blood. Positive and negative reaction controls were included. Amplification products were resolved in a 1.5% agarose gel stained with GelRed ™ (Biotium, ref. 41003, United States) and visualized under UV light. The limit of detection of the nPCR used in the study is 1 p/µL.

### Data management and statistical analysis

All data were collected using standardized questionnaires and forms and entered into a Microsoft Access database and Excel sheet. Data analysis was carried out using SPSS version 23.0. Infection prevalence was derived and 95% confidence interval (CI) was calculated when applicable. Sensitivity, specificity, positive predictive value (PPV) and negative predictive value (NPV) were determined for LM (comparator test), RDT (comparator test) and LAMP (index test), using nPCR as the reference standard. Kappa coefficient was calculated to assess the agreement among different diagnostic methods. Complementary analysis for diagnostic test accuracy was carried out in two subgroups of participants (afebrile and febrile). *p* values < 0.05 were considered statistically significant.

### Ethics

The study was reviewed and approved by the Facultad de Medicina Ethics Committee at the Universidad de Antioquia, Medellín, Colombia (Record 005; 31st March 2016). The study was conducted in accordance with the Declaration of Helsinki and local rules and regulations of Colombia. Before starting any study procedure, written informed consent or an informed assent in the case of women < 18 years were obtained from each participant. Additional consent was obtained for parents or legal guardians of minors.

### Definitions

Low-density infection [43] was defined as *Plasmodium* spp. infection detected by nPCR but not by LM. Febrile infections were defined as infections detected by LM, RDT, LAMP or nPCR in pregnant women with fever (axillary temperature ≥ 37.5 °C) or history of fever in the last 3 days [31]. Afebrile infections were defined as infections detected by LM, RDT, LAMP or nPCR in pregnant women without fever or history of fever in the last 3 days [31]. Febrile pregnant women were defined as study participants with fever (axillary temperature ≥ 37.5 °C) or history of fever in the last 3 days, regardless malaria infection. Afebrile pregnant women were defined as study participants without fever or history of fever in the last 3 days, regardless malaria infection.

## Results

### Baseline characteristics of pregnant women

**Fig. 2:**
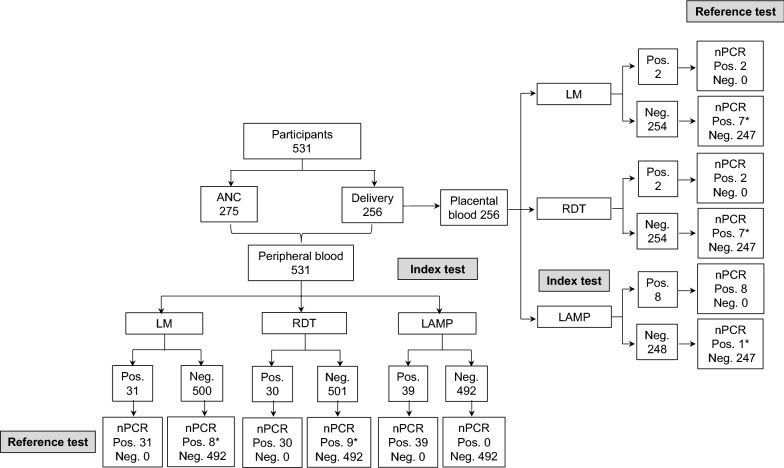
Study participant flow and testing results. The chart shows the total number of pregnant women recruited during antenatal care visits and at delivery, the number of peripheral and placental blood samples collected, as well as the overall number of malaria infections detected by each test. *Discrepant results when compared with the reference test. *ANC* antenatal care, *Pos.* positive, *Neg.* negative, *LM* light microscopy, *RDT* rapid diagnostic test *LAMP* loop-mediated isothermal amplification, *nPCR* nested polymerase chain reaction

A total of 531 pregnant women were enrolled across all study sites (Fig. 2): 91 in El Bagre, 220 in Quibdó and 220 in Tumaco. Overall, 51.8% (275/531) of participants were recruited during ANC visits and 48.2% (256/531) at delivery. Baseline characteristics of study participants are shown in Table 1. Median age of pregnant women was 24 years (age range 15–45) and nearly half of women (49.3%; 262/531) were primigravidae. At enrolment, the prevalence of anaemia was 41.8% (222/531) and only 3.8% (20/531) of pregnant women presented fever or reported history of fever within the last 3 days (8.1%; 43/531). The percentage of participants reporting history of malaria within current pregnancy was 5.5% (29/531).

**Table 1:** Baseline characteristics of participants at enrolment across all study sites

	Total	ANC	Delivery
Total number of participants	531	275	256
Age (years): median (IQR)	24 (20–28)	23 (19–28)	25 (21–29)
Gestational age (weeks): median (IQR)	35 (18–38)	18 (14–26)	38 (37–38)
Gravidity: median (IQR)	1 (0–1)	1 (0–2)	0 (0–1)
Primigravidae: N (%)	262 (49.3)	116 (42.2)	146 (57.0)
Anaemia (haemoglobin < 11 mg/dL): N (%)	222 (41.8)	110 (40.0)	112 (43.7)
Axillary temperature (°C): median (IQR)	36.8 (36.4–37.0)	36.7 (36.3–36.8)	36.8 (36.6–37.1)
Fever at enrolment: N (%)	20 (3.8)	11 (4.0)	9 (3.5)
History of fever last 72 h: N (%)	43 (8.1)	23 (8.4)	20 (7.8)
Malaria history: N (%)			
Malaria within current pregnancy	29 (5.5)	18 (6.6)	11 (4.3)
Anti-malarials taken during current pregnancy	24 (4.7)	13 (4.9)	11 (4.4)

*ANC* antenatal care, *IQR* interquartile range, *N* sample size

### Malaria positivity rate by diagnostic test

In peripheral blood samples (Table 2), the overall malaria positivity rate by LAMP was 7.3% (39/531), the same percentage as the reference test (nPCR). Thirty positive pregnant women were detected by RDT (5.6%; 30/531) and 31 by LM (5.8%; 31/531), with an overall median parasitaemia density of 2480 p/µL (parasitaemia range 80–54582 p/µL). As confirmed by nPCR, the majority of infections (89.7%; 35/39) were caused by *P. falciparum*, while two of these (5.1%; 2/39) were co-infections with *P. vivax*. The prevalence of *P. vivax* mono-infection detected by the reference test was 7.7% (3/39). In addition, one participant (2.6%; 1/39) in the ANC group was infected with *P. malariae*. Eight samples positive by LM were detected during passive surveillance in afebrile women. Low-density infections detected by nPCR (20.5%; 8/39) were identified in afebrile pregnant women, seven of which were caused by *P. falciparum* and one by *P. malariae*.

**Table 2:** Diagnostic test results and prevalence of *Plasmodium* spp. among pregnant women

	LM	RDT	LAMP	nPCR
Peripheral blood (N = 531)				
All pregnant; N (%)				
Total	31 (5.8)	30 (5.6)	39 (7.3)	39 (7.3)
*P. falciparum*	26 (5.1)	26 (5.1)	35 (6.6)	33 (6.2)
*P. vivax*	4 (0.8)	3 (0.6)	3 (0.6)^a^	3 (0.6)
*P. malariae*	0	NA	1 (0.2)^a^	1 (0.2)
*Mixed* (*P. falciparum/P. vivax*)	1 (0.2)	1 (0.2)	NA	2 (0.4)
ANC (N = 275); N (%)				
Total	31 (11.3)	30 (10.9)	34 (12.4)	34 (12.4)
*P. falciparum*	26 (9.4)	26 (9.4)	30 (10.9)	28 (10.2)
*P. vivax*	4 (1.4)	3 (1.1)	3 (1.1)^a^	3 (1.1)
*P. malariae*	0	NA	1 (0.4)^a^	1 (0.4)
*Mixed* (*P. falciparum/P. vivax*)	1 (0.4)	1 (0.4)	NA	2 (0.7)
Delivery (N = 256); N (%)				
Total	0	0	5 (2.0)	5 (2.0)
*P. falciparum*	0	0	5 (2.0)	5 (2.0)
Placental blood (N = 256); N (%)				
Total	2 (0.8)	2 (0.8)	8 (3.1)	9 (3.6)
*P. falciparum*	2 (0.8)	2 (0.8)	8 (3.1)	8 (3.1)
*P. vivax*	0	0	0	1 (0.4)

*ANC* antenatal care, *LM* light microscopy, *RDT* rapid diagnostic test, *LAMP* loop-mediated isothermal amplification, *nPCR* nested polymerase chain reaction, *NA* not applicable, *N* sample size

^a^LAMP Pan positive/LAMP *P. falciparum* negatives; specie confirmed by nPCR

When comparing the total number of *Plasmodium* positive samples in the ANC and delivery group, a higher number of infections were identified during ANC visits detected by nPCR (12.4% (34/275) vs. 1.9% (5/256), p < 0.001) and also with other test [LM = 11.2% (31/275) vs. 0% (0/256); RDT = 10.9% (30/275) vs. 0% (0/256); LAMP = 12.4% (34/275) vs. 1.9% (5/256) 1.9%].

Regarding placental samples (Table 2), LAMP identified eight infections (3.1%; 8/256) and nPCR nine (3.6%; 9/256), while RDT and LM detected approximately one quarter (22.2%; 2/9) of those identified by the reference test. The predominant causative pathogen identified by nPCR in placental blood was *P. falciparum* (88.9%; 8/9).

### Test performance in peripheral blood collected during ANC visits and at delivery

Using nPCR as the reference test, LAMP showed an improved sensitivity [100.0% (95% CI = 92.4–100)] when compared to LM [79.5% (95% CI = 64.5–89.2); *p *< 0.01] and RDT [76.9% (95% CI = 61.7–87.4); *p *< 0.01]. No statistically significant difference was observed between LM and RDT sensitivity. Specificity was similar in all tests [100.0% (95% CI 99.2–100.0)] (Table 3).

**Table 3:** Performance of LM, RDT and LAMP for diagnosing malaria in peripheral blood samples

Test					Value (95% CI)				
		nPCR^a^			Sensitivity	Specificity	PPV	NPV	Kappa
		(+)	(−)	Total					
LM									
(+)		31	0	31	79.5% (64.5–89.2)	100.0% (99.2–100.0)	100.0% (89.0–100.0)	98.4% (96.9–99.2)	0.9 (0.8–1.0)
(−)		8	492	500					
RDT									
(+)		30	0	30	76.9% (61.7–87.4)	100.0% (99.2–100.0)	100.0% (88.6–100.0)	98.2% (96.6–99.1)	0.9 (0.8–1.0)
(−)		9	492	501					
LAMP									
(+)		39	0	39	100.0% (92.4–100.0)	100.0% (99.2–100.0)	100.0% (92.0–100.0)	100.0% (99.2–100.0)	1.00 (1.0–1.0)
(−)		0	492	492					

LM: light microscopy; RDT: rapid diagnostic test; LAMP: loop-mediated isothermal amplification; nPCR: nested polymerase chain reaction; (+): positive; (−): negative; PPV: positive predictive value; NPV: negative predictive value; CI: confidence interval

^a^nPCR was used as the reference test

Discrepant results between LAMP and nPCR were initially observed in six samples: three LAMP positive/PCR negative and three LAMP negative/PCR positive. Those samples were run in triplicate using nPCR and confirmed the original LAMP results.

Considering test performance in febrile (50) and afebrile (437) pregnant women, all methods showed very similar results for detecting febrile infections (23/50). LM and LAMP displayed higher sensitivity [100.0% (CI 95% = 87.5–100.0)] than RDT [95.0% (CI 95% = 76.4–100.0)] (Table 4). Nonetheless, in afebrile cases (16/437), LAMP sensitivity was superior to that of LM and RDT [100.0% (CI 95% = 80.6–100.0) vs. 50.0% (CI 95% = 28.0–72.0%); *p *< 0.01]. Among the 39 malaria positive cases detected by nPCR, the prevalence of febrile and afebrile infections was 59.0% (23/39) and 41.0% (16/39), respectively. The median parasite density in febrile infections was 3235 p/μL (range 370–41,210) and the proportion of low-density infections was 0% (0/23) while in afebrile infections the median parasite density was 1410 (range 140–3540) and the proportion of low-density infections was 52.9% (9/17).

**Table 4:** Accuracy of LM, RDT and LAMP for diagnosing symptomatic and asymptomatic malaria in peripheral blood

	Value (95% CI)				
	Sensitivity	Specificity	PPV	NPV	Kappa
Symptomatic (N = 50)^a^					
LM	100.0% (85.7–100.0)	100.0% (87.5–100.0)	100.0% (85.7–100.0)	98.7% (87.5–100.0)	1.0 (1.0–1.0)
RDT	95.0% (76.4–99.0)	100.0% (87.5–100.0)	100.0% (83.2–100.0)	96.3% (82.3–99.4)	1.0 (0.9–1.0)
LAMP	100.0% (85.7–100.0)	100.0% (87.5–100.0)	100.0% (85.7–100.0)	98.7% (87.5–100.0)	1.0 (1.0–1.0)
Asymptomatic (N = 437)^a^					
LM	50.0% (28.0–72.0)	100.0% (99.1–100.0)	100.0% (67.6–100.0)	98.0% (96.4–99.1)	0.7 (0.4–0.9)
RDT	50.0% (28.0–72.0)	100.0% (99.1–100.0)	100.0% (67.6–100.0)	98.0% (96.4–99.1)	0.7 (0.4–0.9)
LAMP	100.0% (80.6–100.0)	100.0% (99.1–100.0)	100.0% (80.6–100.0)	100.0% (99.1–100.0)	1.0 (1.0–1.0)

*LM* light microscopy, *RDT* rapid diagnostic test, *LAMP* loop-mediated isothermal amplification, *PPV* positive predictive value, *NPV* negative predictive value, *CI* confidence interval, *N* sample size

^a^nPCR was used as the reference test

### Test performance on placental blood collected at delivery

Using nPCR on placental blood as the reference test, LAMP showed a four-fold improvement in sensitivity [88.9% (CI 95% = 56.5–98.0)] when compared with LM and RDT [22.2% (CI 95% = 6.3–54.7)]. Specificity was similar in all tests [100% (95% CI 98.5–100.0)] (Table 5).

**Table 5:** Performance of LM, RDT, LAMP for diagnosing malaria in placental blood samples

Placental blood^a^ (N = 256)	Value (95% CI)		
	Sensitivity	Specificity	Kappa
LM	22.2% (6.3–54.7)	100.0% (98.5–100.0)	0.0 (ND)
RDT	22.2% (6.3–54.7)	100.0% (98.5–100.0)	0.0 (ND)
LAMP	88.9% (56.5–98.0)	100.0% (98.5–100.0)	0.9 (0.8–1.1)

*LM* light microscopy, *RDT* rapid diagnostic test, *LAMP* loop-mediated isothermal amplification, *CI* confidence interval, *N* sample size, *ND* non-determined

^a^nPCR was used as the reference test

The performance of LM, RDT, LAMP and nPCR for the diagnosis of placental malaria in peripheral blood samples was also assessed. When screening peripheral blood obtained at delivery from pregnant women who also provided placental blood (n = 256), no infection was detected by LM or RDT. LAMP and nPCR detected five of the nine placental infections in peripheral blood identified by nPCR on placental blood samples (reference test in this case), translating in a sensitivity of 55.6% (95% CI 26.7–81.0) for both diagnostic techniques (Table 6).

**Table 6:** Performance of LM, RDT, LAMP and nPCR for diagnosing placental malaria in peripheral blood samples

Peripheral blood (N = 256)^a^	Value (95% CI)		
	Sensitivity	Specificity	Kappa
LM	0.0 (ND)	0.0 (ND)	0.0 (ND)
RDT	0.0 (ND)	0.0 (ND)	0.0 (ND)
LAMP	55.6% (26.7–81.0)	100% (98.8–100.0)	0.7 (0.4–1.0)
nPCR	55.6% (26.7–81.0)	100% (98.8–100.0)	0.7 (0.4–1.0)

*LM* light microscopy, *RDT* rapid diagnostic test, *LAMP* loop-mediated isothermal amplification, *nPCR* nested polymerase chain reaction, *CI* confidence interval, *N* sample size, *ND* non-determined

^a^Peripheral blood obtained at delivery from pregnant women who also provided placental blood. nPCR in placental blood was used as the reference test

## Discussion

This is, to our knowledge, the first study evaluating the performance of LAMP for screening malaria in pregnant women in the endemic areas of a low malaria transmission country. Using nPCR as the reference standard, overall results showed an improved sensitivity of LAMP for the diagnosis of gestational and placental malaria compared with LM and RDT. LAMP also showed similar performance to nPCR for detecting low-density parasitaemia, as well as for identifying malaria infections in febrile and afebrile pregnant women. In addition, the study demonstrates that LAMP methodology can be successfully deployed outside a reference laboratory and at local hospitals in malaria endemic areas with minimum infrastructure conditions.

In the current study, the overall MiP positivity rate in peripheral blood confirmed by nPCR was lower (7.3%) than previous studies conducted in Colombia (14–32%) [13, 14], but comparable to recent studies carried out in comparable endemic areas (5.8–8.7%) [2, 44]. The prevalence of malaria in placental samples (3.6%) was also in line with latest studies (3.0%) [2], and lower than in former ones (16.5–37.7%) [13, 14], likely as a result of the intervention measures conducted in the country to decrease malaria burden. The majority of infections reported in the current study were caused by *P. falciparum* (> 80% in both, peripheral and placental blood) [6], particularly in Tumaco and Quibdó, where population is Afro-descendant with high prevalence of Duffy-negative individuals [45].

When using LAMP on peripheral samples, a significant improvement in sensitivity was observed as compared with LM and RDT (100% vs. 79.5 and 76.9%, respectively), and overall agreement among LAMP and nPCR was high (kappa = 1.0). These results were consistent with previous studies proving higher performance of LAMP for the diagnosis of malaria in pregnant [28, 29] and non-pregnant women [12, 21, 23–27] in different endemic settings. Most importantly, LAMP and PCR were able to identify a considerable amount of low-density infections not detected by LM (20%; 8/39). This observation was in agreement with previous studies in MiP in Colombia using PCR [13] and suggested that LM—currently used for passive case detection of malaria infections [6] and recommended by Colombian health authorities for screening of malaria at ANC visits—is not sensitive enough for routine detection of MiP in the country.

Consequently and in light with these results, further studies are needed to evaluate the potential integration of LAMP into clinical and surveillance programs to promptly detect and effectively treat malaria infection in pregnant women attending ANC visits and at delivery. There is also a need to evaluate the costs per assay comparing to conventional test, including equipment, reagents, labour, training and maintenance in order to evaluating the cost-effectiveness of LAMP for their potential integration into clinical and surveillance programmes.

Interestingly, while all methods displayed similar accuracy in symptomatic pregnant women, LAMP showed the highest sensitivity (100%) to detect infections in afebrile participants, two times better than LM and RDT (50%). The clinical relevance of pregnant women as asymptomatic parasite reservoirs is not yet fully understood, but in South American countries only the overall prevalence of asymptomatic MiP accounts for 22% of all malaria cases [46]. In high-transmission settings, several studies correlated asymptomatic *P. falciparum* infections with anaemia [47, 48]. Although recent results indicated that *P. vivax* asymptomatic infections were not associated with an increased risk of maternal anaemia in low-transmission settings [2], asymptomatic carriers could nevertheless constitute reservoirs for malaria transmission [8, 16]. In this regard, the identification and management of asymptomatic malaria cases by highly sensitive tools such as LAMP might be key for malaria elimination programs. Especially, among pregnant women who inherently present low-density infections. Moreover, it is crucial to evaluate the impact that this highly sensitive tool may have on malaria-related adverse outcomes for pregnancy and birth, as well as on malaria transmission.

Regarding placental malaria, LAMP showed a four-fold improvement in sensitivity compared with LM and RDT (22.2%). Similar results have been reported in the literature, showing that LAMP was more sensitive than LM [29, 30], in both asymptomatic and symptomatic cases in pregnant women [30]. Remarkably, the recent study conducted by Kapisi et al. revealed that higher malaria burden in pregnancy was associated with placental malaria and that detection of parasites in this tissue was correlated with an increased risk of adverse birth outcomes [30]. In light with the above findings, the improved sensitivity of LAMP suggests that this method could be an optimal diagnostic test for placental malaria. Indeed, using nPCR as reference test, LAMP had high sensitivity (88.8%) and specificity (100%), only missing one *P. vivax* low-density positive specimen probably due to low parasite density.

Additionally, LAMP could be a useful tool for detecting placental infections in peripheral blood. One of the findings of this study was that approximately half of the placental infections were detected in peripheral blood by LAMP and nPCR, while missed by LM or RDT. This result suggested that most placental infections remain hidden when conventional diagnostic test are used [13, 49, 50], probably because parasites are sequestered in placental tissue and circulate at low-density levels in peripheral blood [9–11]. Further studies are needed to investigate whether maternal peripheral blood might mirror placental infections detected by LAMP but are hindered by the fact that access to placental tissue and blood before delivery is practically impossible.

Last but not least, one of the objectives of the study was to evaluate the feasibility of performing LAMP outside a reference laboratory. As reported in previous studies carried out in field condition [25–27], LAMP was compatible with the routine procedures used in the three local hospitals (i.e. LM, RDT). No major incident was reported during the implementation of the test (e.g. sample contamination, sample degradation), showing that technicians without strong experience in molecular tools who only received a 2-days training were able to conduct LAMP testing in malaria endemic settings with minimum infrastructure. Therefore, the combination of high sensitivity and specificity together with the easiness of use in remote settings, makes LAMP a valuable tool for the detection of MiP in low-transmission areas.

### Study limitations

The main limitation of this study was the low number of malaria-positive samples, particularly for placental blood. The current research was linked to a larger cross-sectional project in which sample size was estimated to meet the main objective of the parental study, but not the current sub-study related to LAMP performance in pregnant women. Despite not being powered, LAMP showed an improved performance detecting *Plasmodium* parasites in pregnant women, when compared to LM and RDT. In addition, it is worth noting that some women (4.7%) reported having received anti-malarial treatment during pregnancy. Lastly, the current format of the LAMP kit does not allow differentiating between *P. falciparum* mixed-species infections and *P. falciparum* mono-species infections. Nevertheless, by combining the Pan-LAMP and Pf-LAMP kits, it was possible to identify all *Plasmodium* infections confirmed by nPCR (*P. falciparum*, *P. vivax* and *P. malariae*). In line with this, a product able to identify all *Plasmodium* spp., particularly *P. vivax*, is needed to guide appropriate malaria treatment in clinical settings.

## Conclusions

LAMP showed an improved sensitivity for the diagnosis of gestational and placental malaria, when compared with LM and RDT and high agreement with nPCR, especially for detecting low-density infections and screening malaria in afebrile cases. LAMP represents a highly sensitive and less complex alternative to PCR-based tests for the detection of MiP outside reference laboratories. Although the clinical relevance of low-parasitaemia afebrile cases needs to be further investigated, the current findings highlight the need to revise the current MiP surveillance system, currently relying on LM and RDTs only. This is particularly true in low-transmission settings, such as the Colombian malaria endemic regions, where intermittent preventive treatment is not recommended. In this context, LAMP could be a valuable tool for active case detection of malaria cases during ANC visits and at delivery, helping to prevent malaria-related pregnancy and birth complications.

## Abbreviations

ANC: antenatal care
CI: confidence interval
DNA: deoxyribonucleic acid
LM: light microscopy
LAMP: loop-mediated isothermal amplification
MiP: malaria in pregnancy
NAATs: nucleic acid amplification techniques
NPV: negative predictive value
p/μL: parasite/μL
PCR: polymerase chain reaction
nPCR: nested polymerase chain reaction
RDT: rapid diagnostic test
spp.: species
PPV: positive predictive value
UV: ultraviolet
